# Insanity and Divorce

**Published:** 1882-09

**Authors:** 


INSANITY AND DIVORCE.—Some time ago a commission was appointed in Paris to investigate the question of Divorce, and determine what circumstances should in future be accepted as valid reasons for the dissolution of the marriage tie. MM. Charcot, Blanche and Legrand du Saulle have recently appeared before this commission, and testified that, with some reservations, insanity should not hold in law as a plea for divorce. According to the *British Medical Journal*, a writer in the *Gazette Hebdomadaire* warmly endorses the opinion of these eminent men, and in support of it advances the following reasons:

1st. No *disease* occurring after marriage, not even impotence, justifies divorce; insanity being due to a diseased condition of the nervous system, can therefore no more justify divorce than any other form of disease. The insane suffer in their moral and intellectual, as well as their physical health, and consequently deserve even greater consideration and care from their friends than those afflicted with ordinary ailments. In many cases, it is true, strict precautions must be taken to guard against the effects of mental loss of balance, but these do not necessitate either divorce or separation.

2d. *Chronic Insanity*, although often incurable, is not necessarily so. It would be both cruel and unjust to make it legally possible for a married man to return to his home after an unexpected recovery from chronic insanity to find his wife divorced from him and wedded to some other man. The form of dementia known in France as *Folie Ciaculaire* often yields such surprises. M. Blanche cites one case of this kind which, after sixteen years recovery eventually took place. *General paralysis* is admittedly incurable; yet such long and complete remissions do sometimes occur as to deceive even the most experienced practioners, the disease seems permanently arrested, and the patient leaves the asylum and returns to his family. With regard to these hopeless cases which are manifestly passing from bad to worse, Charcot maintains that the plain duty of the family is to redouble every possible attention, and wait patiently for the inevitable end, rather than seek for relief by divorce. *Epilepsy*, though generaly continuous, and a fruitful cause of insanity, is nevertheless sometimes limited to two or three attacks, then disappears never to return. According to Foville, such epilepsies occur chiefly in the young, and are most frequently connected with the eruption of the wisdom teeth. Epilepsy is sometimes caused by the irritation of intestinal worms; the case of a librarian is cited where epileptic attacks ceased after the expulsion of a tænia, and did not return during the remaining fifteen years of his life. Chronic insanity should not therefore be admitted as a reason for divorce.

3d. It is by no means uncommon to meet with persons sufficently wanting in the moral sense to speculate by marriage on the diseased condition of their fellow creatures. Those affections which are slow in presenting their true character, but are not unfrequently recognizable in their early stages, such as phthisis and insanity, are particularly adapted to this kind of calculation. The prospect of divorce would be yet another encouragement, and so much the more tempting as regards dementia, that it would be more easy to accomplish an act of spoliation in the case of a person of weak intellect after marriage.— *Canada Med. Rec.*, April.

